# Reduction of Premature Aging Markers After Gastric Bypass Surgery in Morbidly Obese Patients

**DOI:** 10.1007/s11695-018-3247-3

**Published:** 2018-04-25

**Authors:** P. J. Hohensinner, C. Kaun, B. Ebenbauer, M. Hackl, S. Demyanets, D. Richter, M. Prager, J. Wojta, Gersina Rega-Kaun

**Affiliations:** 1 Department of Internal Medicine II, Division of Cardiology, Medical University of Vienna, Waehringer Guertel 18-20, 1090 Vienna, Austria; 2 Ludwig Boltzmann Cluster for Cardiovascular Research, Vienna, Austria; 3 TAmiRNA GmbH, Vienna, Austria; 4 Department of Laboratory Medicine, Medical University of Vienna, Vienna, Austria; 5 Department of General Surgery, Territory Hospital Oberwart, Oberwart, Austria; 6 Department of Surgery, Hospital Hietzing, Vienna, Austria; 7 Core Facilities, Medical University of Vienna, Vienna, Austria; 8 5th Medical Department for Endocrinology and Rheumatology, Wilhelminenhospital, Vienna, Austria

**Keywords:** Premature aging, Bariatric surgery, Telomere length, Telomere oxidation, miRNA, Senescence-associated secretory proteins

## Abstract

**Background:**

Obesity is considered to be a major comorbidity. Obese patients suffer from an increased proinflammatory state associated with a premature aging phenotype including increased secretion of senescence-associated secretory proteins (SASP) and reduced telomere length. Micro-ribonucleic acids (miRNAs) are non-coding RNA molecules that could modify the post-transcriptional process. Several studies have reported associations between miRNAs and metabolic unhealthy conditions.

**Aim:**

To determine if bariatric surgery and the resulting weight loss could reverse the premature aging phenotype.

**Methods:**

We enrolled 58 morbidly obese patients undergoing bariatric surgery. Markers of premature aging including the SASP IL-6, CRP and PAI-1, 7 miRNAs, as well as telomere length and telomere oxidation in mononuclear cells were evaluated.

**Results:**

Patients showed a significant drop of body mass index (BMI; 43.98 ± 3.5 versus 28.02 ± 4.1, *p* < 0.001). We observed a significant reduction in SASP including a reduction of 55% of plasma IL-6 levels (*p* = 0 < 0.001), 83% of CRP levels (*p* = 0.001) and 15% of plasma PAI-1 levels (*p* < 0.001). Telomere length doubled in the patient cohort (*p* < 0.001) and was accompanied by a reduction in the telomere oxidation index by 70% (*p* < 0.001). Telomere length was inversely correlated with telomere oxidation. The aging-associated miRNA miR10a_5p was upregulated significantly (*p* = 0.039), while the other tested miRNAs showed no difference.

**Conclusion:**

Our data indicate a significant reduction of the proinflammatory SASP after bariatric surgery. We observed an increase in telomere length and reduced oxidative stress at telomeres. miR10a_5p which is downregulated during aging was upregulated after surgery. Overall, bariatric surgery ameliorated the premature aging phenotype.

## Introduction

Obesity is becoming a global epidemic and is associated with numerous comorbidities, especially cardiovascular disease and diabetes [1]. Obesity is clinically defined by a body mass index (BMI) above 30 kg/m^2^ [2]. Adipose tissue, recognized now as an endocrine organ, is an extensive source of proinflammatory cytokines and growth factors [3]. Obese patients are therefore thought to be in a permanent state of low-grade inflammation.

Obesity accelerates the aging process. In a prospective cohort study, the Framingham Heart Study, overweight and obesity were associated with a large decrease in life expectancy and an increase in early mortality [4]. In addition, obese patients suffer from an increase in age-associated disease prevalence suggesting a premature aging phenotype [5]. A link between obesity and premature aging is a permanent inflammatory state. In addition, adipose tissue from obese individuals contains higher numbers of senescent cells compared to lean age-matched controls [6, 7]. These cells are characterized by the production of senescence-associated secretory proteins (SASP) [8]. SASP members include interleukin-6 (IL-6), tumor necrosis factor-α (TNF-α), several matrixmetalloproteases (MMPs), and the protease inhibitor plasminogen activator inhibitor-1 (PAI-1), which taken together support the inflammatory state of aging [9]. Besides changes in protein secretion, also circulating levels of micro-ribonucleic acids (miRNAs) that are capable of fine-tuning inflammatory responses have been observed to change with age [10, 11].

Cellular age can be determined by measuring the length of telomeres. Telomeres are the natural ends of chromosomes, protecting the integrity of chromosomal DNA and avoiding replicative loss of vital information at chromosomal ends [12]. Obesity is associated with an acceleration of aging including telomere length reduction [13, 14]. Previous reports already demonstrated reduced telomere length in obese patients compared to lean subjects with the authors suggesting an additional 8.8 years of aging with obesity [13]. Furthermore, an inverse correlation of telomere length and BMI has already been described [15]. Rode et al. have linked a genetically increased BMI with short telomeres and the inflammatory marker C-reactive protein (CRP) [16].

Current guidelines recommend surgical intervention at a BMI > 40 kg/m^2^ or a BMI > 35 kg/m^2^ with secondary disease, a minimum age of 18 years, and the failure or futility of a structured conservative program [17]. After surgery, patients drastically lose weight mainly during the first 1 to 2 years after surgery [18]. This results in already reported reduced levels of proinflammatory cytokines [19]. However, telomeric changes seem to follow different time kinetics. Formichi et al. observed a decrease in telomere length 1 year after bariatric surgery [20]. Laimer et al. in contrast suggested that telomeres elongate in bariatric surgery patients over an investigation period of 10 years [21].

The aim of our study was to evaluate markers of premature aging including SASP and miRNAs, telomere length, and telomere stability in patients before and 2 years after gastric bypass surgery.

## Materials and Methods

### Patient Recruitment and Sampling

Study participants were enrolled after they were selected to undergo Roux-en-Y gastric bypass surgery. Before surgery and at follow-up visits, a venous blood drawing was performed. After centrifugation (2800 rpm, 20 min), plasma and serum samples were stored at − 80 °C in multiple aliquots. Whole blood samples for DNA isolation were collected before surgery and at 24 months, aliquoted and frozen immediately.

### Protein Determination

Cytokine levels were determined using commercially available ELISA kits for high sensitive IL-6 (hs-IL6; R&D Systems, MN, USA) and PAI-1 (Technoclone, Austria) in EDTA plasma samples as suggested by the manufacturers. Concentrations of highly sensitive CRP (hs-CRP) were measured using particle-enhanced immunoturbidimetric assay on cobas® 8000 modular analyzer (Cardiac C-Reactive Protein (Latex) High Sensitive, Roche Diagnostics, Switzerland).

### RNA Extraction

Total RNAs including miRNAs were extracted from exactly 200 μl of EDTA plasma samples using an automated Maxwell system (Promega, WI, USA) with the respective miRNA isolation kit (miRNA tissue lysis kit, Promega) according to the manufacturer’s instructions. A mix of three synthetic spike-in controls (Exiqon, Denmark) was added to the lysis buffer prior to isolation in order to monitor RNA extraction efficiencies.

### miRNA Determination

Circulating miRNA analysis was performed according to standardized protocols for RT-qPCR analysis, as described in several previous publications [22–25]. Briefly, exactly 2 μl total RNA was converted to cDNA using the universal cDNA synthesis kit II (Exiqon) at 42 °C for 60 min, followed by inactivation at 95 °C for 5 min. To every reaction, 1 μl of cel-miR-39 had been added to monitor the efficiency of reverse transcription. cDNA was diluted 1:50 for qPCR reactions. qPCR was set up in 10-μl reactions using ExiLENT SYBR® Green Mastermix (Exiqon) and LNA microRNA primer assays (Exiqon). PCR conditions were 95 °C for 10 min of activation followed by 45 cycles of denaturation (95 °C, 10 s) and annealing/elongation (60 °C, 60 s), and melting curve analysis. PCR amplification and fluorescence detection were performed on a Roche LightCycler 480 II in 96-well plates. The second derivate method was applied to call Cq-values. Cq-values observed for the spike-in controls were used to determine the analytical variation accumulating during RNA extraction, reverse transcription, and qPCR analysis and to check for presence of enzyme inhibitors. RNA spike-in values were used for normalization of miRNA Cq-values to obtain delta-Cq (dCq) values. Hemolysis was checked using the ratio of miR-23a-3p vs. miR-451a, as described previously [26].

### miRNA Screening and Target Selection

Sixteen miRNA were selected due to their previously reported association with aging (Table 1). Seven miRNA (in gray) were selected after the screening in a total of eight patients according to their lowest *p* values. For the screening, these eight patients were selected as they had weight loss and hs-CRP reduction closest to the overall cohort average. In detail, patients were selected according to the following calculation:

$$ score= abs\left(\frac{\left( weight(A)- weight(B)\right)- AWL}{AWL}\ast \frac{\left( CRP(A)- CRP(B)\right)- ACD}{ACD}\right) $$

**Table 1:**
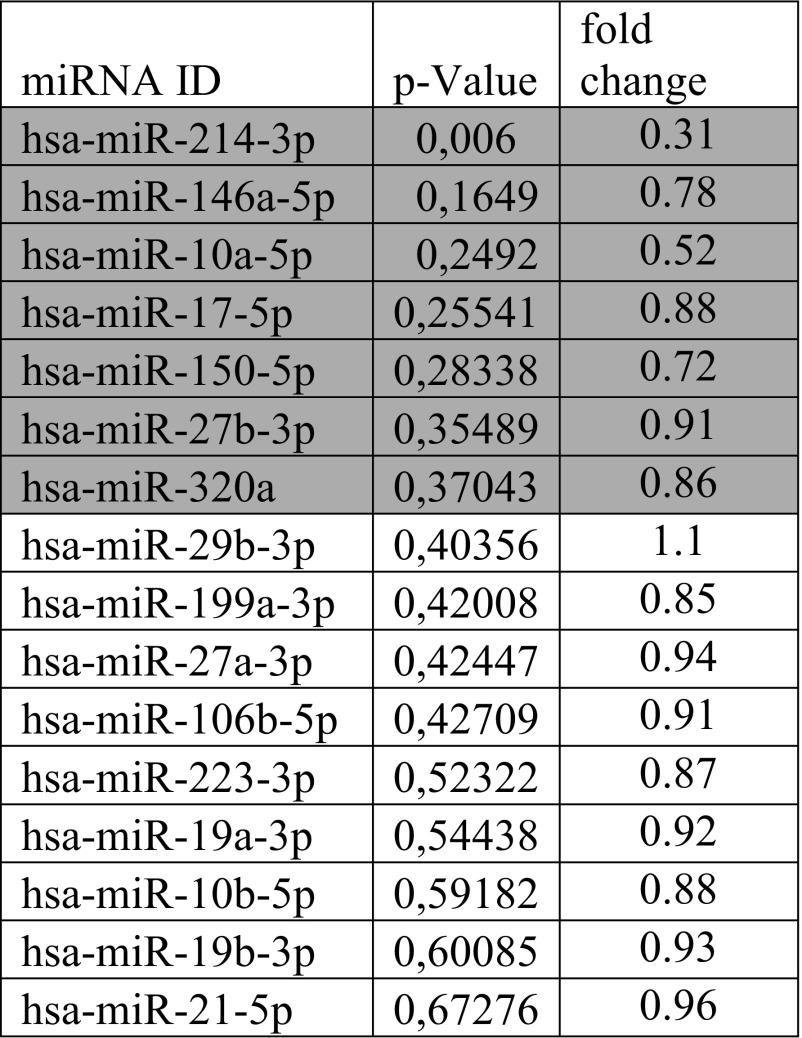
miRNA screening

Aging-associated miRNA selected for an initial screen in eight patients. miRNA in gray were selected to be determined in all 51 available patients

A: pre surgery
B: 2 years post-surgery
AWL: average weight loss of all patients
ACD: average difference of CRP levels of all patients.

### DNA Isolation

DNA was isolated from whole blood samples using a Maxwell automated system together with the respective Blood DNA kit (both Promega).

### Measurement of Relative Telomere Length

We used a qPCR-based method as published previously [27], which is similar to the one used by Laimer et al. [21]. In short, a telomere-specific primer (forward primer 5′-cggtttgtttgggtttgggtttgggtttgggtttgggtt-3′, reverse primer 5′-ggcttgccttacccttacccttacccttacccttaccct-3′) was used to determine relative telomeric length using a GoTaq Real Time PCR system (Promega). Input was normalized to the single copy gene 36B4 (forward primer 5′-cagcaagtgggaaggtgtaatcc-3′, reverse primer 5′-cccattctatcatcaacgggtacaa-3′) run under the same conditions. qPCR was performed on a LightCycler 480 System (Roche, Switzerland) using LightCycler software (Roche). Cycling conditions for both targets consisted of an initial 95 °C step for 10 min followed by 50 cycles of 95 °C for 15 s and 60 °C for 1 min. All samples were run at the same time for a total of two times. Delta C_T_ method was used to calculate results; results are given in arbitrary units (a.u.).

### Measurement of Oxidized Telomeres

Oxidized telomeres were measured as described previously [27, 28]. The telomere sequence is rich in guanine bases which are also prone to oxidation leading to 8-oxoguanine lesions. These lesions can be cleaved specifically using the enzyme FPG (NEB, MA, USA). Isolated DNA was incubated with or without FPG enzyme for 16 h at 37 °C followed by a relative telomere length determination by qPCR. Due to sequence cleavage at 8-oxoguanine bases, qPCR signals are reduced in the enzymatically digested sample. The delta C_T_ difference of undigested to digested signal is calculated and expressed as percent control and normalized to the telomere length of the specific donor. Results are given as telomere oxidation normalized to telomere length in arbitrary units.

### Statistics

Statistical calculations were performed using SPSS 21 (IBM, NY, USA). Changes between time points were calculated by Wilcoxon ranks test with significance assumed at *p* ≤ 0.05 after testing parameters with a Kolmogorov-Smirnov test. Correlations are given by the Pearson correlation factor R and are considered significant at *p* ≤ 0.05. Differences between time points were calculated by subtracting the post-surgery value from the pre-surgery value. miRNA results were determined by a log transformation of corrected Cq-values as suggested for percentage change ratios [29] followed by a paired Student’s *t* test.

## Results

The patient cohort consisted of 17 male (29%) and 41 female patients with a mean age of 41.9 ± 11.2 years. Baseline and follow-up characteristics 2 years after bariatric surgery are depicted in Table 2. Eighteen patients (31%) presented with diabetes. Patients drastically lost weight and hence had a massive reduction of BMI after surgery.

**Table 2:** Baseline characteristics of 58 patients before and 2 years after bariatric surgery

	Before surgery	2 years after surgery	*p* value
	*n* = 58		
Weight (kg)	127.36 ± 17.19	81.43 ± 16.68	< 0.001
BMI	43.98 ± 3.55	28.02 ± 4.08	< 0.001
Total Cholesterol (mg/dl)	178.57 ± 37.61	161.52 ± 35.54	0.001
Triglyceride (mg/dl)	156.71 ± 144.38	84.4 ± 31.86	< 0.001
Medication			
Statins	14 (24.1%)	3 (5.2%)	
Antidiabetics	14 (24.1%)	4 (6.9%)	
Insulin	4 (6.9%)	3 (5.2%)	
ACE inhibitors	26 (44.8%)	13 (22.4%)	
Beta blockers	15 (25.9%)	4 (6.9%)	

*BMI* body mass index

Values are given as mean ± standard deviation, *p* values were calculated using Wilcoxon rank test

**Fig. 1:**
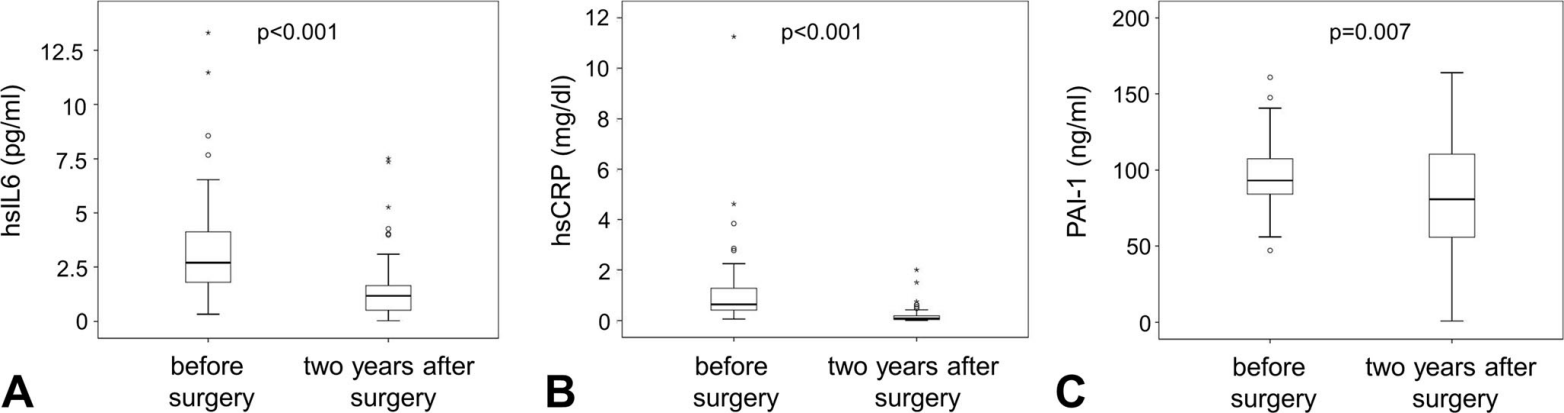
Proinflammatory senescence-associated secretory proteins before and after bariatric surgery. Plasma levels for the SASP proteins IL-6 (a), CRP (**b**), and PAI-1 (**c**) were determined in samples obtained before and 2 years after bariatric surgery as indicated in Materials and Methods. All three protein levels dropped significantly after bariatric surgery. Circulating plasma level concentrations are given on the respective *y*-axis. Samples were compared using Wilcoxon rank test. *p* < 0.05 was considered statistically significant

When analyzing the inflammatory mediator and SASP IL-6, we found that 2 years after bariatric surgery, hs-IL-6 dropped significantly by 55% from a mean of 3.2 ± 2.4 pg/ml before surgery to 1.5 ± 1.5 pg/ml after surgery (*p* < 0.001, Fig. 1a). Concordantly, hs-CRP levels were significantly reduced by 83% from 1.1 ± 1.6 mg/dl before surgery to 0.2 ± 0.3 mg/dl after surgery (*p* < 0.001, Fig. 1b). At both time points, hs-IL6 and hs-CRP correlated significantly (*R* = 0.560, *p* < 0.001 before surgery and *R* = 0.715, *p* < 0.001 after surgery). We found a significant albeit not drastic reduction of 15% from 97.8 ± 22.6 ng/ml before surgery to 83.1 ± 41.4 ng/ml 2 years after surgery for the adipokine and SASP PAI-1 (*p* = 0.007, Fig. 1c). Weight difference did not correlate with differences measured at protein levels for IL6 and CRP but correlated slightly with differences in PAI (*R* = 0.261, *p* = 0.047). Of note, diabetic patients presented with slightly higher PAI-1 levels (93.3 ± 20.2 ng/ml for non-diabetic patients and 108.1 ± 24.9 ng/ml for diabetic patients, *p* = 0.02). However, this association was lost after surgery. Hs-IL6 and hs-CRP starting and end values as well as differences for all three proteins before and after surgery did not show a difference for diabetic and non-diabetic patients.

**Fig. 2:**
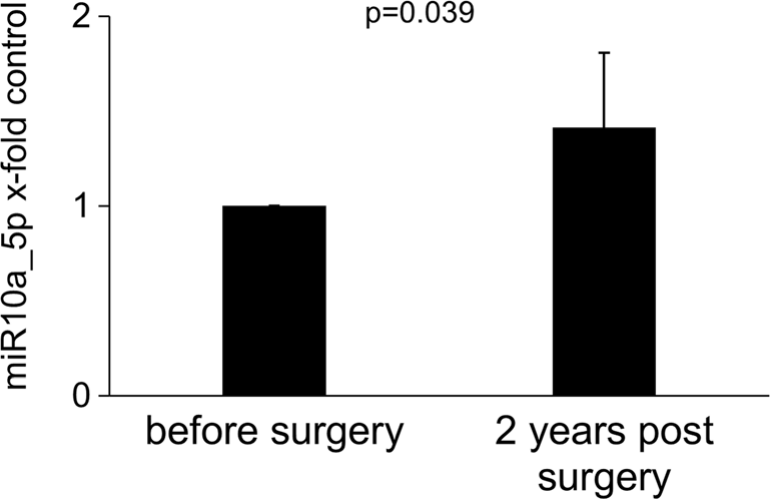
Levels of miR10a_5p before and 2 years after bariatric surgery. Circulating miRNAs were determined in plasma samples from 51 patients before and 2 years after bariatric surgery. Values for miR10a_5p are given as x-fold over baseline. Statistical significance was determined using paired *t* test as indicated in Materials and Methods and *p* < 0.05 was considered significant

To identify changes in the miRNA signature of patients before and after bariatric surgery, we initially analyzed 16 miRNA targets in a subset of 8 patients (Table 1 and Materials and Methods for selection criteria). From this screen, seven targets with the lowest *p* values were selected to be determined in all 51 available patients. We only found a statistically significant increase for miR10a_5p with the remaining tested miRNAs showing no significant change 2 years after surgery. For simplicity, fold change for miR10a_5p is depicted in Fig. 2.

**Fig. 3:**
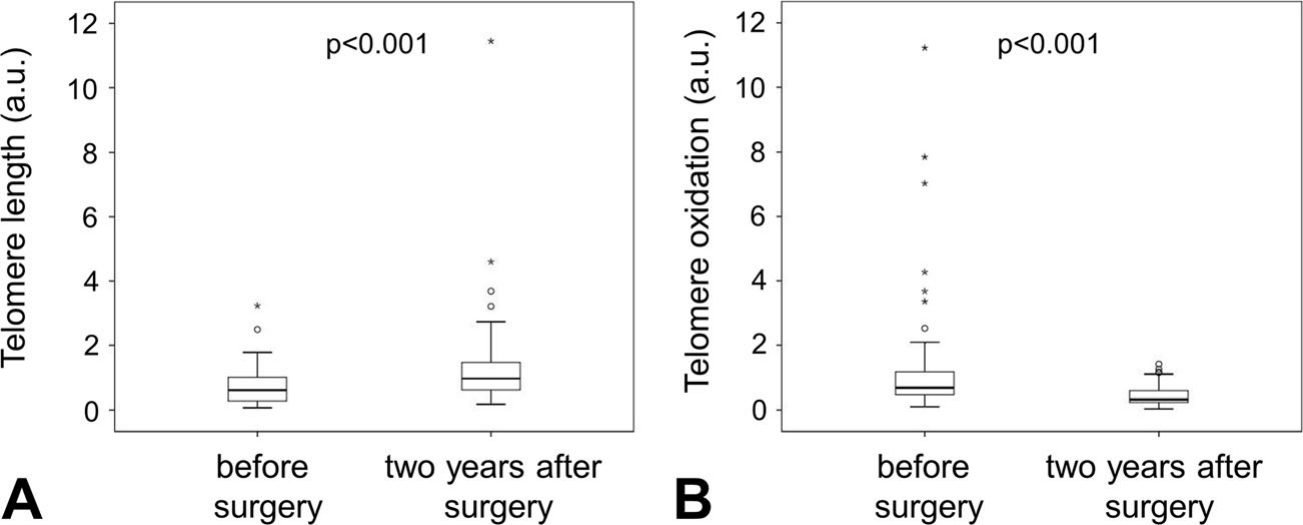
Telomere length and telomere oxidation in bariatric surgery patients before and 2 years after surgery. Telomere length (**a**) was measured via qPCR as indicated in Materials and Methods in patients before and 2 years after bariatric surgery. We found a significant increase of relative telomere length after surgery. In addition, telomeres displayed reduced oxidation damage determined as described in Materials and Methods (**b**) indicating a more telomere protective environment. Statistical significance was determined using Wilcoxon rank test. *p* < 0.05 was considered statistically significant

Telomere length was determined 24 months after bariatric surgery. Telomere length doubled from initially 0.74 ± 0.6 to 1.6 ± 0.2 a.u. 24 months after surgery (*p* < 0.001, Fig. 3a). Telomere oxidation dropped significantly by more than two thirds from 1.3 ± 1.9 to 0.4 ± 0.3 a.u. (*p* < 0.001, Fig. 3b). At both time points, we observed a negative correlation for telomere length and telomere oxidation (*R* = − 0.458, *p* < 0.001 before surgery and *R* = − 0,367, *p* = 0.005 after surgery). Again, diabetic patients did not show different behavior regarding telomere length or telomere oxidation changes. However, increased weight change was directly associated with increased telomere length (*R* = 0.287, *p* = 0.029).

## Discussion

In this study, we show that dramatic weight loss in morbidly obese patients is accompanied by a reduction of the SASP IL-6 and PAI-1 and the inflammatory marker and acute phase reactant CRP confirming previous data [30, 31]. In addition, we describe circulating premature aging-related miRNAs before and after bariatric surgery. Furthermore, we demonstrate that weight loss in these patients is directly associated with increased telomere length and we show for the first time that this might be caused by a reduction of telomere oxidative stress.

Bariatric surgery is the most effective treatment for severe obesity [32]. A key feature of gastric bypass surgery is that it allows the patients to maintain a markedly reduced body weight in the long term [33]. Effects of bariatric surgery are, however, not limited to weight loss alone, but include improvement of adipose tissue function, reduction in proinflammatory adipokines, and changes in insulin sensitivity [34]. In line with previous results, we found here a marked decrease in proinflammatory markers IL-6 and CRP in patients 2 years after bariatric surgery together with a reduction of the adipokine and SASP PAI-1. Telomere length after 2 years increased significantly within the patient cohort together with a significant drop of telomere oxidation, suggesting a reduction if not reversal of premature aging in those patients.

We investigated the two SASP-associated factors IL-6 and PAI-1 in plasma in this patient cohort. Both proteins are induced in obese patients but are also expressed increasingly at baseline in aging cells without prior stimulation [9, 35]. IL-6 as well as PAI-1 showed a significant drop in plasma after bariatric surgery. PAI-1 reduction correlated with weight loss, which might be a considerable factor for the decrease in PAI-1 plasma levels, as adipose tissue is considered to be the main source of increased PAI-1 in obese individuals [36, 37]. It would be tempting to speculate that at least part of this reduction in PAI-1 originates from the loss of aged cells in the adipose tissue. Our hypothesis would be supported by the notion that adipose tissue is a site of considerable senescent cell accumulation [7], which would vanish after bariatric surgery. Similar factors could be influential for reduced plasma levels of IL-6, especially as subcutaneous adipose tissue is associated with increased IL-6 production [38] even though IL-6 reduction was not directly associated with the degree of weight loss in our cohort.

A hallmark of premature aging is an increased overall stress for cells and organs. This stress in form of sterile inflammation or increased oxidative stress might have detrimental consequences for the telomere length of individual cells. We measured the telomere length of circulating cells from the bloodstream. This cell population will have been replenished several times over the time course of 2 years. However, letting the immune system accustom to a more stable environment might explain why we were able to observe an increase in telomere length, whereas Formichi described telomeric loss after 1 year [20], especially as the most dramatic weight loss and hence stress is happening within the first year. In addition, we observed a dramatic reduction of 8-oxoguanine lesions on the telomere. Future investigations are needed to clarify whether the telomere elongation observed after bariatric surgery is due to an actual increase in telomere length or due to a reduction of oxidative stress on the telomere and therefore a reduction in telomere end breaking.

A panel of seven circulating miRNAs with known associations to inflammation and aging were determined by qPCR in a total of 51 patients. We found an increase in miR10a while the other tested miRNAs showed no significant change. Previously, miR10a was described to be downregulated in aging of mesenchymal stem cells and to be involved in the reduced differentiation capacity of aged mesenchymal stem cells [39]. In addition, changes might be more pronounced at earlier time-points following surgery. Ortega et al. reported a difference in miR21 1 year after bariatric surgery [40]. Changed miRNA circulating profiles have been so far mainly linked with severe diseases including patients presenting with neurological disorders including Alzheimer’s disease or Parkinson’s disease [41, 42]. Of note Ameling et al. described that out of 155 miRNAs, only 12 were associated with aging in a healthy population-based cohort indicating only a limited involvement of circulating miRNA in aging [43].

In conclusion, we demonstrate for the first time an increase of telomere length associated with a reduced telomere oxidation after bariatric surgery. We speculate that the reduction of proinflammatory cytokines presented in our study could ameliorate the oxidative burden and thus could lead to a reduced premature aging phenotype after massive weight loss.
